# Poly[tetraaqua-μ_4_-squarato-di-μ_3_-squarato-disam­arium(III)]

**DOI:** 10.1107/S160053680803674X

**Published:** 2008-11-20

**Authors:** Hocine Akkari, Sofiane Bouacida, Patricia Bénard-Rocherullé, Hocine Merazig, Thierry Roisnel

**Affiliations:** a Département de Chimie, Faculté des Sciences, Université du 20 août 1955-Skikda, route d′El-Hadaïk, BP 26, 21000 Skikda, Algeria; b Département de Chimie, Faculté des Sciences et Sciences de l’Ingénieur, Université A. Mira de Béjaia, Route Targua Ouzmour 06000 Béjaia, Algeria; c Sciences Chimiques de Rennes (UMR CNRS 6226), Université de Rennes 1, Avenue du Général Leclerc, 35042 Rennes Cedex, France; d Laboratoire de Chimie Moléculaire, du Contrôle de l’Environnement et de Mesures Physico-Chimiques, Département de Chimie, Faculté des Sciences Exactes, Université Mentouri 25000 Constantine, Algeria

## Abstract

The structure of the title compound, [Sm_2_(C_4_O_4_)_3_(H_2_O)_4_]_*n*_, consists of infinite-chain structural units, built from edge-sharing samarium SmO_7_(H_2_O)_2_ polyhedra and linked *via* bis-monodendate squarate (sq1) groups. The chains extend along [100] in a zigzag mode and are interconnected by bis-chelating squarate (sq2) ligands into layers parallel to (101). Inter­layer hydrogen bonds strengthen the cohesion of the three-dimensional network. The samarium cation is coordinated by four O atoms from sq1 units and three O atoms from sq2 units, in addition to two water O atoms. The best representation of the samarium SmO_7_(H_2_O)_2_ polyhedron is distorted tricapped trigonal-prismatic. The sq1 ligand has one metal-free O atom and relates three Sm atoms in a bis-monodentate and chelation fashion, the second squarate, sq2, is strictly centrosymmetric and acts as a bis-chelating ligand.

## Related literature

For lanthanide squarates, see: Trombe *et al.* (1988 ▶, 1990 ▶); Petit *et al.* (1990 ▶).
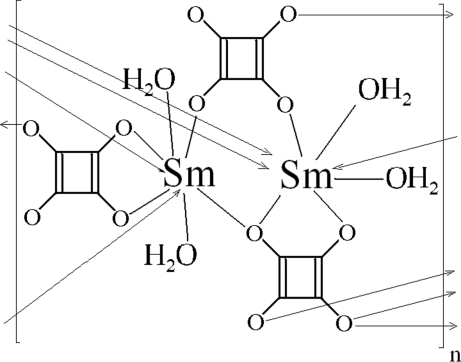


## Experimental

### 

#### Crystal data

[Sm_2_(C_4_O_4_)_3_(H_2_O)_4_]
*M* *_r_* = 708.88
Monoclinic, 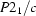

*a* = 7.0824 (1) Å
*b* = 16.7725 (3) Å
*c* = 6.9066 (1) Å
β = 101.585 (1)°
*V* = 803.72 (2) Å^3^
*Z* = 2
Mo *K*α radiation
μ = 7.33 mm^−1^
*T* = 296 (2) K
0.15 × 0.14 × 0.14 mm

#### Data collection

Nonius KappaCCD diffractometer
Absorption correction: none
3906 measured reflections
2340 independent reflections
2199 reflections with *I* > 2σ(*I*)
*R*_int_ = 0.029

#### Refinement

*R*[*F* ^2^ > 2σ(*F* ^2^)] = 0.023
*wR*(*F* ^2^) = 0.058
*S* = 1.08
2340 reflections
148 parameters
6 restraints
H atoms treated by a mixture of independent and constrained refinement
Δρ_max_ = 1.54 e Å^−3^
Δρ_min_ = −1.96 e Å^−3^

### 

Data collection: *COLLECT* (Nonius, 1998 ▶); cell refinement: *SCALEPACK* (Otwinowski & Minor, 1997 ▶); data reduction: *DENZO* (Otwinowski & Minor, 1997 ▶) and *SCALEPACK*; program(s) used to solve structure: *SIR2002* (Burla *et al.*, 2003 ▶); program(s) used to refine structure: *SHELXL97* (Sheldrick, 2008 ▶); molecular graphics: *ORTEP-3* (Farrugia, 1997 ▶) and *DIAMOND* (Brandenburg & Berndt, 2001 ▶); software used to prepare material for publication: *WinGX* (Farrugia, 1999 ▶).

## Supplementary Material

Crystal structure: contains datablocks global, I. DOI: 10.1107/S160053680803674X/dn2395sup1.cif

Structure factors: contains datablocks I. DOI: 10.1107/S160053680803674X/dn2395Isup2.hkl

Additional supplementary materials:  crystallographic information; 3D view; checkCIF report

## Figures and Tables

**Table 1:** Hydrogen-bond geometry (Å, °)

*D*—H⋯*A*	*D*—H	H⋯*A*	*D*⋯*A*	*D*—H⋯*A*
O2*W*—H2*W*2⋯O2^i^	0.94 (4)	1.78 (4)	2.716 (3)	173 (4)
O1*W*—H1*W*1⋯O2^ii^	0.92 (2)	1.97 (3)	2.882 (3)	171 (4)
O2*W*—H1*W*2⋯O6^iii^	0.94 (4)	1.96 (4)	2.865 (3)	162 (4)
O1*W*—H2*W*1⋯O3^i^	0.94 (4)	1.90 (4)	2.834 (3)	179 (5)

Symmetry codes: (i) 
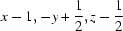
; (ii) 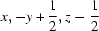; (iii) 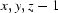.

**Fig. 1.**
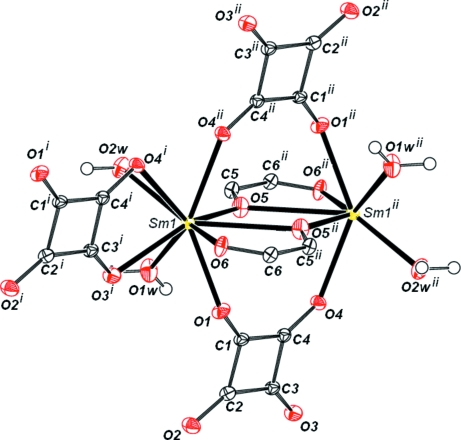
View of the molecular geometry of the title compound with the atom-labelling scheme. Displacement ellipsoids are drawn at the 50% probability level. H atoms are represented as small spheres of arbitrary radii. [Symmetry codes: (i) x-1, y, z; (ii) -x, -y+1, -z+1]

**Fig. 2.**
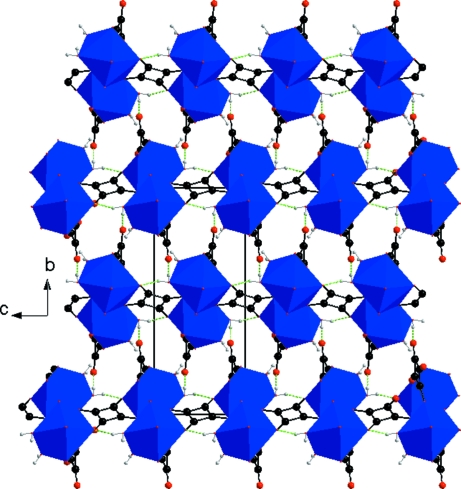
A diagram of the layered crystal packing in the unit cell of (I). Hydrogen bonds are shown as green dashed lines.

## supplementary crystallographic information

### Comment

A family of weakly hydrated lanthanide(III) squarates, [Ln(H_2_O)_2_]_2_ (C_4_O_4_)_3 _ (Ln= La, Ce, Pr, Nd, Sm, Eu) was synthesized hydrothermally (Trombe *et al.*, 1988; Trombe *et al.*, 1990) starting from heating hydrated lanthanide(III) squarates (Petit *et al.*, 1990) in water inside a closed vessel. Unit-cell parameters of the whole compounds were determined from X-ray powder patterns. However, only crystal structure of cerium compound was determined using single-crystal X-ray techniques. Accordingly, much effort was given to experimental conditions in order to obtain single-crystals of the other lanthanides. Unfortunately, up to now no other single-crystal of lanthanide(III) squarate tetrahydrates could be obtained. Therefore herein we report on crystal structure of Di(samarium(III)diaqua) trisquarate, [Sm(H_2_O)_2_]_2_(C_4_O_4_)_3_ (I).

The compound (I) was synthesized during one of our attempts to create novel lanthanide(III) squarates. However, only [Sm(H_2_O)_2_]_2_(C_4_O_4_)_3_ separated from solution and a view of the molecular structure is given in Fig. 1. Trombe *et al.* reported the structure of cerium(III) squarate tetrahydrate (Trombe *et al.*, 1988; Trombe *et al.*, 1990). The samarium analog is isostructural, the compound crystallizing with similar unit-cell dimensions. Its molecular structure displays a layered structure based on infinite chains structural units, built from edge sharing distorted tricapped trigonal prism polyhedra SmO_7_(H_2_O)_2_ (Fig. 2). These polyhedra are connected *via* regular squarate sq1 groups along [100] in zigzag mode and interconnected by bis-chelating squarate anions sq2 into layers parallel to (101) plan (Fig. 2). Hydrogen bonds strengthen the structure (table 1, Fig. 2). The two crystallographically independent squarate anions present a case of chelation. One (sq1) has no imposed symmetry. One of its oxygen atoms, namely O2, is not bound to any samarium atom. The oxygen atoms O3 and O4 chelate one samarium atom. The atom O4 binds also another samarium atom while the atom O3 binds only one samarium atom. Thus, the squarate sq1 ligand relates three metal centers. The second symmetric squarate sq2 ligand chelates on both side one samarium atom, and one more Sm atom is also bounded to the oxygen atom O5 of each bite , so that four metal centers are related.

### Experimental

For convenience, 3,4-dihydroxy-3-cyclobutene-1,2-dione (H_2_C_4_O_4_) is named squaric acid hereafter. Pale yellow single crystals of the title compound were hydrothermally synthesized during an attempt to synthesize open frameworks of lanthanide squarates. A mixture of samarium chloride, SmCl_3_.6H_2_O, and squaric acid in molar ratio 2/3/704 were dissolved in 10 ml distilled water while stirring. The resulting mixture (pH = 2) was transferred into was transferred into a Teflon-lined acid digestion bomb (Parr) and heated at 150 °C for two days under autogenous pressure. Then the autoclave was cooled to room temperature by turning off the power. The pH after treatment remains unchanged. Products were filtered off, washed with distilled water and dried at room temperature. Crystals with cubic morphology were selected for single-crystal diffraction after checking under a polarizing microscope and identifying by X-ray powder diffraction.

### Refinement

All non-H atoms were refined with anisotropic atomic displacement parameters. All H atoms were localized on Fourier maps and refined isotropically with soft constraints on the distances to their relevant parent water oxygen atom. Within a molecule, the O–H and H–H distances were restrained to 0.96 Å and 1.55 Å, respectively, so that the H–O–H angle fitted the ideal value of the tetrahedral angle.

### Crystal data

**Table:**

[Sm_2_(C_4_O_4_)_3_(H_2_O)_4_]	*F*_000_ = 664
*M**_r_* = 708.88	*D*_x_ = 2.929 Mg m^−^^3^
Monoclinic, *P*2_1_/*c*	Mo *K*α radiation λ = 0.71073 Å
Hall symbol: -P 2ybc	Cell parameters from 5454 reflections
*a* = 7.0824 (1) Å	θ = 2.6–42.1º
*b* = 16.7725 (3) Å	µ = 7.33 mm^−^^1^
*c* = 6.9066 (1) Å	*T* = 296 (2) K
β = 101.585 (1)º	Cube, yellow
*V* = 803.72 (2) Å^3^	0.15 × 0.14 × 0.14 mm
*Z* = 2	

### Data collection

**Table:**

Nonius KappaCCD diffractometer	2199 reflections with *I* > 2σ(*I*)
Monochromator: graphite	*R*_int_ = 0.029
*T* = 296(2) K	θ_max_ = 30.0º
φ scans, and ω scans with κ offsets	θ_min_ = 2.9º
Absorption correction: none	*h* = −9→9
3906 measured reflections	*k* = −23→22
2340 independent reflections	*l* = −9→9

### Refinement

**Table:**

Refinement on *F*^2^	Secondary atom site location: difference Fourier map
Least-squares matrix: full	Hydrogen site location: difference Fourier map
*R*[*F*^2^ > 2σ(*F*^2^)] = 0.023	H atoms treated by a mixture of independent and constrained refinement
*wR*(*F*^2^) = 0.058	*w* = 1/[σ^2^(*F*_o_^2^) + (0.103*P*)^2^ + 0.3358*P*] where *P* = (*F*_o_^2^ + 2*F*_c_^2^)/3
*S* = 1.08	(Δ/σ)_max_ = 0.002
2340 reflections	Δρ_max_ = 1.54 e Å^−^^3^
148 parameters	Δρ_min_ = −1.96 e Å^−^^3^
6 restraints	Extinction coefficient: ?
Primary atom site location: structure-invariant direct methods	

### Special details

**Table:**

Geometry. Bond distances, angles *etc*. have been calculated using the rounded fractional coordinates. All su's are estimated from the variances of the (full) variance-covariance matrix. The cell e.s.d.'s are taken into account in the estimation of distances, angles and torsion angles
Refinement. Refinement of *F*^2^ against ALL reflections. The weighted *R*-factor *wR* and goodness of fit *S* are based on *F*^2^, conventional *R*-factors *R* are based on *F*, with *F* set to zero for negative *F*^2^. The threshold expression of *F*^2^ > σ(*F*^2^) is used only for calculating *R*-factors(gt) *etc*. and is not relevant to the choice of reflections for refinement. *R*-factors based on *F*^2^ are statistically about twice as large as those based on *F*, and *R*- factors based on ALL data will be even larger.

### Fractional atomic coordinates and isotropic or equivalent isotropic displacement parameters (Å^2^)

**Table:**

	*x*	*y*	*z*	*U*_iso_*/*U*_eq_	
Sm1	−0.26067 (2)	0.42004 (1)	0.48851 (2)	0.0098 (1)	
O1	0.0020 (3)	0.34943 (13)	0.6725 (3)	0.0183 (6)	
O1W	−0.1467 (3)	0.32226 (14)	0.2658 (4)	0.0255 (7)	
O2	0.2263 (3)	0.18597 (13)	0.6602 (3)	0.0207 (6)	
O2W	−0.4770 (3)	0.41711 (14)	0.1649 (3)	0.0208 (6)	
O3	0.5958 (3)	0.29019 (13)	0.5489 (3)	0.0183 (6)	
O4	0.3785 (3)	0.44362 (12)	0.5380 (3)	0.0154 (5)	
O5	−0.0375 (3)	0.48117 (13)	0.3160 (3)	0.0183 (6)	
O6	−0.2690 (3)	0.43098 (13)	0.8510 (3)	0.0166 (6)	
C1	0.1656 (3)	0.33329 (16)	0.6427 (4)	0.0129 (7)	
C2	0.2704 (4)	0.25667 (17)	0.6407 (4)	0.0134 (7)	
C3	0.4325 (4)	0.30194 (16)	0.5902 (4)	0.0134 (7)	
C4	0.3272 (4)	0.37486 (16)	0.5872 (4)	0.0127 (7)	
C5	−0.0241 (4)	0.48952 (17)	0.1381 (4)	0.0135 (7)	
C6	−0.1238 (4)	0.46752 (17)	0.9388 (4)	0.0135 (7)	
H2W2	−0.587 (5)	0.385 (3)	0.161 (7)	0.0500*	
H1W1	−0.025 (3)	0.315 (3)	0.241 (7)	0.0500*	
H1W2	−0.422 (7)	0.411 (3)	0.053 (5)	0.0500*	
H2W1	−0.233 (5)	0.285 (2)	0.196 (7)	0.0500*	

### Atomic displacement parameters (Å^2^)

**Table:**

	*U*^11^	*U*^22^	*U*^33^	*U*^12^	*U*^13^	*U*^23^
Sm1	0.0083 (1)	0.0107 (1)	0.0115 (1)	0.0004 (1)	0.0045 (1)	0.0009 (1)
O1	0.0085 (8)	0.0229 (11)	0.0246 (11)	0.0029 (7)	0.0059 (7)	0.0039 (9)
O1W	0.0188 (10)	0.0266 (12)	0.0334 (13)	−0.0030 (9)	0.0111 (9)	−0.0144 (10)
O2	0.0163 (9)	0.0158 (10)	0.0320 (12)	0.0001 (8)	0.0094 (9)	0.0051 (9)
O2W	0.0163 (10)	0.0300 (12)	0.0162 (10)	−0.0024 (8)	0.0033 (8)	−0.0031 (8)
O3	0.0103 (8)	0.0179 (10)	0.0293 (11)	0.0016 (7)	0.0100 (8)	0.0042 (8)
O4	0.0128 (9)	0.0122 (9)	0.0221 (10)	−0.0009 (7)	0.0057 (7)	0.0031 (8)
O5	0.0218 (10)	0.0253 (11)	0.0093 (9)	−0.0052 (8)	0.0069 (7)	−0.0004 (8)
O6	0.0117 (9)	0.0258 (11)	0.0127 (10)	−0.0063 (8)	0.0034 (7)	−0.0032 (8)
C1	0.0094 (10)	0.0133 (12)	0.0164 (12)	0.0022 (9)	0.0034 (9)	0.0018 (10)
C2	0.0116 (11)	0.0165 (12)	0.0130 (12)	0.0002 (9)	0.0046 (9)	0.0044 (10)
C3	0.0101 (10)	0.0147 (12)	0.0158 (12)	0.0014 (9)	0.0037 (9)	0.0020 (10)
C4	0.0102 (10)	0.0137 (12)	0.0143 (12)	0.0001 (9)	0.0030 (9)	0.0014 (9)
C5	0.0130 (11)	0.0180 (13)	0.0099 (11)	−0.0019 (9)	0.0034 (9)	−0.0008 (9)
C6	0.0126 (11)	0.0176 (12)	0.0107 (11)	−0.0007 (9)	0.0033 (9)	0.0003 (10)

### Geometric parameters (Å, °)

**Table:**

Sm1—O1	2.351 (2)	O5—C5	1.259 (3)
Sm1—O1W	2.491 (2)	O6—C6	1.245 (4)
Sm1—O2W	2.443 (2)	O1W—H2W1	0.94 (4)
Sm1—O5	2.393 (2)	O1W—H1W1	0.92 (2)
Sm1—O6	2.523 (2)	O2W—H2W2	0.94 (4)
Sm1—O3^i^	2.474 (2)	O2W—H1W2	0.94 (4)
Sm1—O4^i^	2.675 (2)	C1—C2	1.486 (4)
Sm1—O4^ii^	2.429 (2)	C1—C4	1.456 (4)
Sm1—O5^ii^	2.808 (2)	C2—C3	1.476 (4)
O1—C1	1.247 (3)	C3—C4	1.431 (4)
O2—C2	1.241 (4)	C5—C6^iii^	1.463 (4)
O3—C3	1.261 (4)	C5—C6^ii^	1.457 (4)
O4—C4	1.276 (3)		
			
Sm1···Sm1^iv^	4.3507 (2)	O6···O1	2.833 (3)
Sm1···O2W^iv^	4.293 (2)	O6···O5^ii^	3.036 (3)
Sm1···O2^v^	4.266 (2)	O6···C3^i^	3.297 (3)
Sm1···H2W1^vi^	3.72 (4)	O6···C6^xii^	3.335 (4)
O1···O1W	2.832 (3)	O6···O4^i^	2.964 (3)
O1···O2	3.178 (3)	O6···C5^ii^	2.456 (4)
O1···O3^i^	3.001 (3)	O6···O2W^xiii^	2.865 (3)
O1···O6	2.833 (3)	O6···O2W^iv^	3.108 (3)
O1···C6	2.959 (4)	O6···O3^i^	3.169 (3)
O1···O5^ii^	2.852 (3)	O1···H2W1^vi^	2.83 (3)
O1···C5^ii^	2.992 (4)	O1···H1W1^vi^	2.81 (5)
O1···O1W^vi^	3.176 (3)	O1W···H1W2	2.65 (5)
O1W···C5	3.116 (4)	O2···H2W2^viii^	1.78 (4)
O1W···C6^iii^	3.348 (4)	O2···H1W1^vi^	1.97 (3)
O1W···O1	2.832 (3)	O2W···H2W1	2.79 (3)
O1W···O2W	2.799 (3)	O3···H2W1^viii^	1.90 (4)
O1W···O3^i^	2.978 (3)	O4···H2W2^ix^	2.84 (5)
O1W···O5	2.778 (3)	O5···H1W1	2.84 (5)
O1W···C1	3.064 (4)	O6···H1W2^xiii^	1.96 (4)
O1W···O1^vii^	3.176 (3)	C1···C5^ii^	3.569 (4)
O1W···O2^vii^	2.882 (3)	C2···C2^vi^	3.461 (4)
O1W···O3^v^	2.834 (3)	C2···O3^vi^	3.357 (3)
O2···O1W^vi^	2.882 (3)	C2···O2^vii^	3.409 (3)
O2···O2W^viii^	2.716 (3)	C2···O2W^viii^	3.407 (4)
O2···C3^vi^	3.042 (3)	C2···C3^vi^	3.239 (4)
O2···O1	3.178 (3)	C2···C2^vii^	3.461 (4)
O2···Sm1^viii^	4.266 (2)	C3···O2^vii^	3.042 (3)
O2···C4^vi^	3.066 (3)	C3···C2^vii^	3.239 (4)
O2···C2^vi^	3.409 (3)	C4···O2^vii^	3.066 (3)
O2W···O6^iii^	2.865 (3)	C5···C5^xi^	2.035 (4)
O2W···O2^v^	2.716 (3)	C5···O5^xi^	3.289 (3)
O2W···C2^v^	3.407 (4)	C5···C1^ii^	3.569 (4)
O2W···Sm1^iv^	4.293 (2)	C6···C6^xii^	2.093 (4)
O2W···O4^ii^	3.095 (3)	C6···O2W^xiii^	3.314 (4)
O2W···O1W	2.799 (3)	C6···O6^xii^	3.335 (4)
O2W···O4^i^	2.991 (3)	C6···O1W^xiii^	3.348 (4)
O2W···C6^iv^	3.382 (4)	C6···O2W^iv^	3.382 (4)
O2W···C6^iii^	3.314 (4)	C1···H1W1^vi^	2.97 (4)
O2W···O6^iv^	3.108 (3)	C1···H1W1	2.85 (5)
O3···C2^vii^	3.357 (3)	C2···H2W2^viii^	2.58 (5)
O3···O6^ix^	3.169 (3)	C2···H1W1^vi^	2.62 (3)
O3···O1^ix^	3.001 (3)	C3···H2W1^viii^	2.75 (4)
O3···O4	2.992 (3)	C5···H1W2	3.06 (5)
O3···O1W^ix^	2.978 (3)	C5···H1W1	3.01 (5)
O3···O1W^viii^	2.834 (3)	C6···H1W2^xiii^	2.58 (5)
O4···O5^ii^	3.069 (3)	H2W2···O2^v^	1.78 (4)
O4···O3	2.992 (3)	H2W2···C2^v^	2.58 (5)
O4···O5	3.101 (3)	H1W1···C1	2.85 (5)
O4···O6^ix^	2.964 (3)	H1W1···C5	3.01 (5)
O4···O2W^ix^	2.991 (3)	H1W1···O1^vii^	2.81 (5)
O4···O4^x^	2.679 (3)	H1W1···O2^vii^	1.97 (3)
O4···O2W^ii^	3.095 (3)	H1W1···C1^vii^	2.97 (5)
O5···O6^ii^	3.036 (3)	H1W1···C2^vii^	2.62 (3)
O5···C4^ii^	3.322 (4)	H1W2···O6^iii^	1.96 (4)
O5···O5^ii^	2.569 (3)	H1W2···C5	3.06 (5)
O5···O1^ii^	2.852 (3)	H1W2···C6^iii^	2.58 (5)
O5···C5^xi^	3.289 (3)	H1W2···H2W1	2.59 (6)
O5···C4	3.379 (4)	H2W1···H1W2	2.59 (6)
O5···O4^ii^	3.069 (3)	H2W1···Sm1^vii^	3.72 (4)
O5···C1^ii^	3.270 (3)	H2W1···O1^vii^	2.83 (3)
O5···O1W	2.778 (3)	H2W1···O3^v^	1.90 (4)
O5···O4	3.101 (3)	H2W1···C3^v^	2.75 (4)
O6···C4^i^	3.208 (4)		
			
O1—Sm1—O1W	71.51 (8)	O4^ii^—Sm1—O5^ii^	72.21 (7)
O1—Sm1—O2W	140.08 (7)	Sm1—O1—C1	132.91 (18)
O1—Sm1—O5	87.41 (7)	Sm1^ix^—O3—C3	109.09 (17)
O1—Sm1—O6	70.97 (7)	Sm1^ix^—O4—C4	103.35 (17)
O1—Sm1—O3^i^	76.87 (7)	Sm1^ii^—O4—C4	139.42 (19)
O1—Sm1—O4^i^	132.68 (7)	Sm1^ix^—O4—Sm1^ii^	116.89 (8)
O1—Sm1—O4^ii^	137.53 (7)	Sm1—O5—C5	136.09 (19)
O1—Sm1—O5^ii^	66.44 (7)	Sm1—O5—Sm1^ii^	121.45 (8)
O1W—Sm1—O2W	69.11 (8)	Sm1^ii^—O5—C5	101.94 (17)
O1W—Sm1—O5	69.29 (7)	Sm1—O6—C6	109.72 (18)
O1W—Sm1—O6	137.37 (8)	Sm1—O1W—H1W1	129 (3)
O1W—Sm1—O3^i^	73.72 (7)	Sm1—O1W—H2W1	120 (2)
O1W—Sm1—O4^i^	127.78 (7)	H1W1—O1W—H2W1	111 (4)
O1W—Sm1—O4^ii^	136.25 (8)	Sm1—O2W—H1W2	118 (3)
O1W—Sm1—O5^ii^	112.32 (7)	H2W2—O2W—H1W2	113 (4)
O2W—Sm1—O5	84.76 (7)	Sm1—O2W—H2W2	114 (3)
O2W—Sm1—O6	140.71 (7)	C2—C1—C4	89.5 (2)
O2W—Sm1—O3^i^	86.17 (7)	O1—C1—C2	132.2 (2)
O2W—Sm1—O4^i^	71.35 (7)	O1—C1—C4	138.2 (3)
O2W—Sm1—O4^ii^	78.88 (7)	O2—C2—C3	137.9 (3)
O2W—Sm1—O5^ii^	136.44 (7)	C1—C2—C3	88.3 (2)
O5—Sm1—O6	127.76 (7)	O2—C2—C1	133.5 (3)
O3^i^—Sm1—O5	142.75 (7)	C2—C3—C4	90.9 (2)
O4^i^—Sm1—O5	138.06 (7)	O3—C3—C2	140.0 (3)
O4^ii^—Sm1—O5	79.07 (7)	O3—C3—C4	129.0 (3)
O5—Sm1—O5^ii^	58.56 (7)	O4—C4—C3	127.0 (3)
O3^i^—Sm1—O6	78.72 (7)	O4—C4—C1	141.7 (3)
O4^i^—Sm1—O6	69.46 (7)	C1—C4—C3	91.3 (2)
O4^ii^—Sm1—O6	86.00 (7)	O5—C5—C6^ii^	127.7 (3)
O5^ii^—Sm1—O6	69.21 (6)	O5—C5—C6^iii^	140.7 (3)
O3^i^—Sm1—O4^i^	70.92 (7)	C6^iii^—C5—C6^ii^	91.6 (2)
O3^i^—Sm1—O4^ii^	134.03 (7)	C5^xiii^—C6—C5^ii^	88.4 (2)
O3^i^—Sm1—O5^ii^	137.16 (6)	O6—C6—C5^xiii^	141.1 (3)
O4^i^—Sm1—O4^ii^	63.11 (7)	O6—C6—C5^ii^	130.5 (3)
O4^i^—Sm1—O5^ii^	119.80 (6)		
			
O1W—Sm1—O1—C1	49.0 (2)	O5—Sm1—O4^ii^—C4^ii^	−10.5 (3)
O2W—Sm1—O1—C1	58.6 (3)	O6—Sm1—O4^ii^—C4^ii^	119.2 (3)
O5—Sm1—O1—C1	−20.1 (3)	O1—Sm1—O5^ii^—Sm1^ii^	102.06 (11)
O6—Sm1—O1—C1	−151.7 (3)	O1—Sm1—O5^ii^—C5^ii^	−70.85 (18)
O3^i^—Sm1—O1—C1	125.9 (3)	O1W—Sm1—O5^ii^—Sm1^ii^	45.64 (12)
O4^i^—Sm1—O1—C1	173.7 (2)	O1W—Sm1—O5^ii^—C5^ii^	−127.27 (18)
O4^ii^—Sm1—O1—C1	−90.8 (3)	O2W—Sm1—O5^ii^—Sm1^ii^	−37.02 (15)
O5^ii^—Sm1—O1—C1	−76.7 (3)	O2W—Sm1—O5^ii^—C5^ii^	150.07 (17)
O1—Sm1—O5—C5	126.2 (3)	O5—Sm1—O5^ii^—Sm1^ii^	0.00 (10)
O1—Sm1—O5—Sm1^ii^	−63.81 (10)	O5—Sm1—O5^ii^—C5^ii^	−172.9 (2)
O1W—Sm1—O5—C5	55.0 (3)	O6—Sm1—O5^ii^—Sm1^ii^	179.65 (12)
O1W—Sm1—O5—Sm1^ii^	−135.00 (12)	O6—Sm1—O5^ii^—C5^ii^	6.74 (17)
O2W—Sm1—O5—C5	−14.6 (3)	Sm1—O1—C1—C4	50.2 (5)
O2W—Sm1—O5—Sm1^ii^	155.38 (10)	Sm1—O1—C1—C2	−125.6 (3)
O6—Sm1—O5—C5	−170.4 (3)	Sm1^ix^—O3—C3—C4	5.9 (4)
O6—Sm1—O5—Sm1^ii^	−0.42 (14)	Sm1^ix^—O3—C3—C2	179.9 (3)
O3^i^—Sm1—O5—C5	62.0 (3)	Sm1^ix^—O4—C4—C3	−6.7 (3)
O3^i^—Sm1—O5—Sm1^ii^	−128.01 (10)	Sm1^ii^—O4—C4—C1	6.3 (6)
O4^i^—Sm1—O5—C5	−69.0 (3)	Sm1^ix^—O4—C4—C1	178.9 (3)
O4^i^—Sm1—O5—Sm1^ii^	101.01 (11)	Sm1^ii^—O4—C4—C3	−179.2 (2)
O4^ii^—Sm1—O5—C5	−94.3 (3)	Sm1—O5—C5—C6^iii^	−2.1 (6)
O4^ii^—Sm1—O5—Sm1^ii^	75.72 (10)	Sm1—O5—C5—C6^ii^	177.5 (2)
O5^ii^—Sm1—O5—C5	−170.0 (3)	Sm1^ii^—O5—C5—C6^ii^	6.2 (3)
O5^ii^—Sm1—O5—Sm1^ii^	−0.02 (12)	Sm1^ii^—O5—C5—C6^iii^	−173.4 (4)
O1—Sm1—O6—C6	63.89 (19)	Sm1—O6—C6—C5^xiii^	−172.7 (3)
O1W—Sm1—O6—C6	93.4 (2)	Sm1—O6—C6—C5^ii^	8.8 (4)
O2W—Sm1—O6—C6	−146.84 (18)	O1—C1—C2—C3	178.7 (3)
O5—Sm1—O6—C6	−7.0 (2)	C4—C1—C2—O2	−174.0 (3)
O3^i^—Sm1—O6—C6	143.7 (2)	O1—C1—C2—O2	3.2 (5)
O4^i^—Sm1—O6—C6	−142.6 (2)	O1—C1—C4—O4	−2.9 (7)
O4^ii^—Sm1—O6—C6	−79.85 (19)	O1—C1—C4—C3	−178.5 (3)
O5^ii^—Sm1—O6—C6	−7.37 (18)	C2—C1—C4—O4	174.0 (4)
O1—Sm1—O3^i^—C3^i^	138.84 (19)	C2—C1—C4—C3	−1.6 (2)
O1W—Sm1—O3^i^—C3^i^	−146.90 (19)	C4—C1—C2—C3	1.5 (2)
O2W—Sm1—O3^i^—C3^i^	−77.56 (18)	O2—C2—C3—C4	173.6 (4)
O5—Sm1—O3^i^—C3^i^	−153.71 (17)	C1—C2—C3—O3	−176.9 (4)
O6—Sm1—O3^i^—C3^i^	65.99 (18)	O2—C2—C3—O3	−1.7 (6)
O1—Sm1—O4^i^—C4^i^	−43.5 (2)	C1—C2—C3—C4	−1.6 (2)
O1W—Sm1—O4^i^—C4^i^	56.25 (19)	O3—C3—C4—C1	177.7 (3)
O2W—Sm1—O4^i^—C4^i^	98.70 (17)	C2—C3—C4—O4	−175.0 (3)
O5—Sm1—O4^i^—C4^i^	157.38 (16)	O3—C3—C4—O4	1.2 (5)
O6—Sm1—O4^i^—C4^i^	−78.46 (17)	C2—C3—C4—C1	1.6 (2)
O1—Sm1—O4^ii^—C4^ii^	63.3 (3)	O5—C5—C6^iii^—O6^iii^	0.9 (7)
O1W—Sm1—O4^ii^—C4^ii^	−54.2 (3)	O5—C5—C6^ii^—O6^ii^	1.2 (5)
O2W—Sm1—O4^ii^—C4^ii^	−97.3 (3)		

Symmetry codes: (i) *x*−1, *y*, *z*; (ii) −*x*, −*y*+1, −*z*+1; (iii) *x*, *y*, *z*−1; (iv) −*x*−1, −*y*+1, −*z*+1; (v) *x*−1, −*y*+1/2, *z*−1/2; (vi) *x*, −*y*+1/2, *z*+1/2; (vii) *x*, −*y*+1/2, *z*−1/2; (viii) *x*+1, −*y*+1/2, *z*+1/2; (ix) *x*+1, *y*, *z*; (x) −*x*+1, −*y*+1, −*z*+1; (xi) −*x*, −*y*+1, −*z*; (xii) −*x*, −*y*+1, −*z*+2; (xiii) *x*, *y*, *z*+1.

### Hydrogen-bond geometry (Å, °)

**Table:**

*D*—H···*A*	*D*—H	H···*A*	*D*···*A*	*D*—H···*A*
O2W—H2W2···O2^v^	0.94 (4)	1.78 (4)	2.716 (3)	173 (4)
O1W—H1W1···O2^vii^	0.92 (2)	1.97 (3)	2.882 (3)	171 (4)
O2W—H1W2···O6^iii^	0.94 (4)	1.96 (4)	2.865 (3)	162 (4)
O1W—H2W1···O3^v^	0.94 (4)	1.90 (4)	2.834 (3)	179 (5)

Symmetry codes: (v) *x*−1, −*y*+1/2, *z*−1/2; (vii) *x*, −*y*+1/2, *z*−1/2; (iii) *x*, *y*, *z*−1.
